# Factors Affecting the Transition from Paper to Digital Data Collection for Mobile Tuberculosis Active Case Finding in Low Internet Access Settings in Pakistan

**DOI:** 10.3390/tropicalmed7080201

**Published:** 2022-08-22

**Authors:** Christina Mergenthaler, Jake D. Mathewson, Abdullah Latif, Hasan Tahir, Vincent Meurrens, Andreas van Werle, Aamna Rashid, Muhammad Tariq, Tanveer Ahmed, Farah Naureen, Ente Rood

**Affiliations:** 1 KIT Royal Tropical Institute Center for Applied Spatial Epidemiology, 1092 AD Amsterdam, The Netherlands; 2 National Tuberculosis Program of Pakistan, (Common Management Unit-HIV, TB, Malaria, Ministry of NHSR&C, Government of Pakistan), F-Block, EPI Building, Prime Minister’s Health Complex, Chak Shahzad, Islamabad 44000, Pakistan; 3 Mercy Corps Pakistan, 189/190, Street 6, 1-9/2 Islamabad, Islamabad 44000, Pakistan; 4 EPCON, 2000 Antwerp, Belgium

**Keywords:** CAPI, PAPI, tuberculosis, active case finding, data quality, digital tools

## Abstract

Between September 2020 and March 2021, Mercy Corps piloted hybrid digital (CAPI) and paper-based (PAPI) data collection as part of its tuberculosis (TB) active case finding strategy. Data were collected using CAPI and PAPI at 140 TB chest camps in low Internet access areas of Punjab and Khyber Pakhtunkhwa provinces in Pakistan. PAPI data collection was performed primarily during the camp and entered using a tailor-performed CAPI tool after camps. To assess the feasibility of this hybrid approach, quality of digital records were measured against the paper “gold standard”, and user acceptance was evaluated through focus group discussions. Completeness of digital data varied by indicator, van screening team, and month of implementation: chest camp attendees and pulmonary TB cases showed the highest CAPI/PAPI completeness ratios (1.01 and 0.96 respectively), and among them, all forms of TB diagnosis and treatment initiation were lowest (0.63 and 0.64 respectively). Vans entering CAPI data with high levels of completeness generally did so for all indicators, and significant differences in mean indicator completeness rates between PAPI and CAPI were observed between vans. User feedback suggested that although the CAPI tool required practice to gain proficiency, the technology was appreciated and will be better perceived once double entry in CAPI and PAPI can transition to CAPI only. CAPI data collection enables data to be entered in a more timely fashion in low-Internet-access settings, which will enable more rapid, evidence-based program steering. The current system in which double data entry is conducted to ensure data quality is an added burden for staff with many activities. Transitioning to a fully digital data collection system for TB case finding in low-Internet-access settings requires substantial investments in M&E support, shifts in data reporting accountability, and technology to link records of patients who pass through separate data collection stages during chest camp events.

## 1. Introduction

As handheld digital devices and data collection tools become more accessible in areas with low Internet access, health outreach initiatives are weighing the advantages and challenges to transitioning from paper and pencil interviewing (PAPI) to computer-aided personal interviewing using digital tools including phones and tablets (CAPI) [1]. CAPI data collection enables researchers to create an organized digital repository of standardized data which are available shortly after entry in a single secured source [2,3,4,5,6]. Digital data that are available in a timelier manner offer a variety of programmatic and analytical advantages, including the capacity for real time monitoring and evaluation and subsequent program re-steering based upon need [7,8,9,10,11,12]. Studies have shown that switching from PAPI to CAPI can also improve program and cost efficiency by reducing time required for data entry and management. It can reduce the chance of human error, leading to improved data quality as measured through accuracy, completeness and internal consistency [8,13,14,15,16,17]. Although digital literacy can vary in low resource settings, pilot programs have yielded positive results when newer users have used CAPI for health outreach, also showing improvements in utilization and perception in a matter of days and weeks beyond the initial training [17,18,19].

Recording data on paper forms is still a common practice among health outreach implementers in low-resource settings due to its familiarity and ease of use, cultural acceptance, and the negative suspicions of the security of digital data [20,21]. The administration of household surveys is still very common during routine surveillance of community health workers, and in particular, in active case-finding for tuberculosis (TB) and other outbreak prone diseases [22,23,24,25]. Adoption of CAPI technology is also hampered by sub-optimal or poor phone and Internet network coverage, which can pose challenges to digital data entry requiring linked forms, even amongst experienced users [23]. These challenges are commonplace in Pakistan, where monitoring and evaluation (M&E) for TB active case-finding (ACF) contends with widespread poor Internet service issues, further compounded by local power outages and security threats, making digital data capture and syncing to a server more difficult [24,26]. In Pakistan, TB ACF is part of the National TB Program’s (NTP) private sector engagement strategy to provide TB services to hard-to-reach populations, and Mercy Corps is one of the key private sector partners conducting ACF to help bridge the TB case detection gap in more remote communities [27].

Using paper to collect data in the field is especially common in TB ACF settings due to the unique workflow of TB chest camps, which requires attendees to move through multiple locations where data collection, and hence form linkage, occurs. This process reflects the TB diagnostic cascade and involves the verbal screening stage, sputum collection stage, X-ray examination stage, and hours or days later, the laboratory procedures. Data collected at each stage must be linked to the correct individual’s record. This can be accomplished digitally if individual records are immediately uploaded to a server and chest camp attendees carry with them a unique ID, barcode, or QR code which can be scanned at each stage. This unique identifier is used to later link data entered at various stages of the cascade in the server database. While this method of digital data collection and linkage has been successfully implemented in a variety of settings, it requires substantial reallocations in technical, logistical, financial, and human resources which many implementers may not be fully equipped or ready to invest in when there is a desire to make the digital shift. Many CAPI tools have been developed to function fully offline, such as the OpenDataKit suite of tools and REDCap, but these do not entirely meet the needs of TB chest camp implementers in remote areas. The various stages of data collection, in which data on individual camp attendees must be entered at different times and locations, and the requirement for a unique ID to link these data throughout different stages, require a sophisticated data management process and tools to facilitate this process [28].

For the above reasons, some TB ACF implementers have started to transition all or elements of data collection from PAPI to CAPI. Hybrid approaches have been effective in largescale maternal and child health surveys, household health surveys, and facility-based data collection pilots, enabling some data elements to be digitized, while keeping other data elements paper-based for a variety of reasons [14,29]. As competing priorities did not enable a full digital transition during implementation, Mercy Corps piloted a hybrid PAPI/CAPI approach to data collection for a subset of its TB chest camps in 2020 and 2021, using a customized CAPI tool available on both Android and desktop devices. Mercy Corps made this decision in order to analyze its chest camp results on a more frequent basis than its traditional yearly analysis, with the aim of steering chest camps to locations which show a high yield of people with TB and have other TB risk factors.

Mercy Corps’s shift toward ACF digitization is also reflective of the paradigm shift adopted by Pakistan’s National TB Program (NTP) to analyze and understand TB burden at the subnational level. TB decision-makers globally are increasingly recognizing the value of having more local, context specific TB burden and epidemiology profiles to prioritize case detection. In Pakistan as in many other LMICs, the wealth of data resulting from active case-finding often goes under-utilized and perhaps “over-aggregated” to high administrative levels, creating challenges in understanding which localities to target for case-finding [30].

With these advances in mind, Mercy Corps piloted a hybrid PAPI/CAPI approach to chest camp data collection between August 2020 and March 2021. Our primary research objective was to compare the quality of data entered in the respective (matching) CAPI and PAPI patient-based records by evaluating completeness, accuracy, and consistency of both datasets. Our secondary objective was to evaluate users’ perceptions on feasibility and sustainability of the hybrid PAPI/CAPI approach and full CAPI approach in low Internet chest camp settings.

## 2. Methods

### 2.1. Study Setting

**Figure 1:**
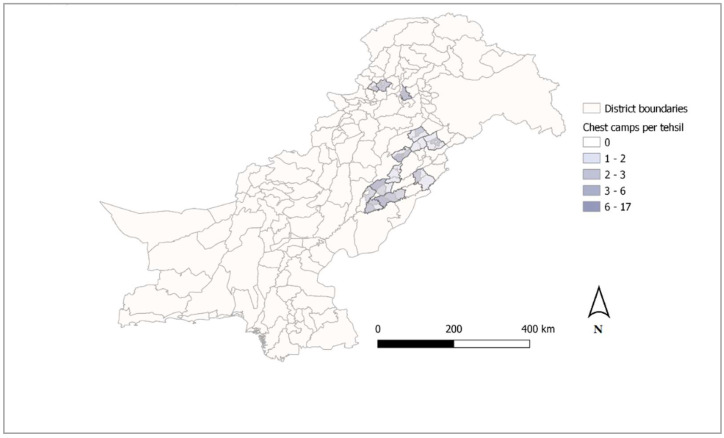
Intervention Area for Hybrid CAPI/PAPI Data Collection in Chest Camps, Pakistan (September 2020–March 2021).

TB ACF was conducted through mobile chest camps in Punjab and Khyber Pakhtunkhwa provinces of Pakistan between August 2020 and March 2021. A map of all tehsils in which chest camps were conducted is shown in Figure 1. Chest camps were operated by a team consisting of at least one community health worker, one clinician, one chest X-ray technician, one district field supervisor (DFS), and one medical doctor in a remote or under-served location. For each chest camp, the team set up a verbal screening station, a medical doctor in-depth screening station, sputum collection station, and a chest X-ray station contained in a van. Chest camp attendees identified as presumptive for TB during verbal screening with the DFS and the medical doctor were invited to provide a sputum specimen and to receive a chest X-ray by the technician working in the van. Sputum specimens were transported to a nearby public sector laboratory for diagnosis, and the DFS called attendees the following day to communicate laboratory results and initiate TB treatment.

### 2.2. Tool Development

To implement a hybrid PAPI/CAPI data collection approach, technical assistance providers to Mercy Corps developed a customized offline-functional Android compatible CAPI tool to capture the essential individual level indicators also captured in the PAPI forms already in use by Mercy Corps, and exact locations of the chest camp which could be optionally captured. An overview and descriptions of the various data collected for these indicators are presented in Table 1. More than 20 DFSs who were responsible for coordination and management of chest camps were trained in at least one half-day session on the installation and use of the CAPI tool application, including digitally entering data in the format of their paper tools. These trainings covered how the CAPI and PAPI data fields mirrored each other, and the skip logic programmed in the CAPI tool, which, for example, did not enable downstream data fields to be populated until earlier fields were entered (i.e., presumptive status selected before chest X-ray or laboratory result could be entered). DFSs were also provided with field guides and instructional videos on YouTube to allow them to troubleshoot in the field or to intermittently refresh their skills.

To assess user acceptance and perceptions on feasibility of using the CAPI tool as part of a hybrid PAPI/CAPI approach, we developed a topic guide for focus group discussions in English comparing perceptions on user-friendliness, efficiency, and safety of both CAPI and PAPI tools. The topic guide was translated into Urdu for the focus group discussions by a local consultant.

### 2.3. Data Collection

#### Quantitative Data Collection

**Figure 2:**
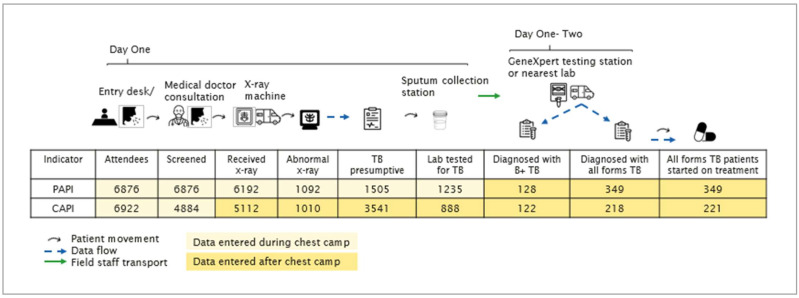
Chest camp intervention logic and flow of participants and data.

Teams operating chest camps entered all individual-level data using both CAPI and PAPI tools (Figure 2). One DFS entered participant registration, verbal symptoms, and TB history using the CAPI tool; in parallel, a second DFS recorded the same attendee registration and symptoms data on paper using individual-level forms. For the remaining stages, data entry during chest camps proceeded on paper (PAPI) according to the pre-existing protocol; i.e., sputum collection was documented on paper and digital chest X-ray results were automatically populated in a desktop-based data repository. Due to the full schedule of a chest camp, digital data entry of data on sputum collection through treatment initiation was performed after the chest camp by re-entering PAPI records into the CAPI tool on the same evening or the following day when teams returned to a stable Internet setting and data could be synced to the Pakistan-based NTP-hosted server. Chest camp data were collected using both PAPI and the tailor-made CAPI tool beginning on 25 August 2020. Use of CAPI paused for several weeks in December, tapering off in February 2021. Paper forms were aggregated to chest camp level into a Microsoft Excel database on a monthly basis. DFS also optionally captured exact locations of the chest camps using the CAPI tool.

### 2.4. Qualitative Data Collection

After obtaining informed consent, the local consultant conducted three 90 min long focus group discussions (FGDs) using the Urdu topic guide with six DFSs each, all of whom had conducted chest camps using the hybrid CAPI/PAPI approach. Each of the five vans (teams) was represented in at least one of the FGDs. FGDs were recorded and transcribed first into Urdu and then into English by the local consultant. Each DFS entered data using CAPI and PAPI tools, either at the verbal screening station or following the chest camp event to retrospectively fill in data.

### 2.5. Data Analysis

We assessed feasibility of the hybrid PAPI/CAPI approach using a mixed methods approach. We assessed CAPI data quality quantitatively by comparing CAPI and PAPI aggregate chest camp data; assessed user acceptance as measured through FGD responses; and triangulated quantitative and qualitative results.

### 2.6. Quantitative Analysis

While our study utilized chest camp data, we referred to the conceptual framework to evaluate digital technology uptake in survey settings to address our research aim, as the framework’s indicators on data completeness, accuracy, and consistency translated well to our study design [14]. We extracted CAPI individual level data for the period of 25 August 2020 through 27 March 2021 from the project server for analysis. To assess quality of data entered into the CAPI tool, we aggregated CAPI data to chest camp level to compare with chest camp level PAPI data. Although PAPI is subject to human error, PAPI chest camp records were assumed to be the “gold standard” for the analysis of data quality, as DFSs entering data were very familiar with the paper forms.

CAPI and PAPI records also contained van team, geographic location, and date variables, which were used for stratified analysis. The numbers of chest camp events and event indicators shown in Table 1 were compared between PAPI and CAPI datasets. All data management and analysis were carried out in Stata version 15.0 SE. Three dimensions of CAPI data quality were assessed by comparing CAPI records with PAPI: completeness, accuracy, and consistency. PAPI records were viewed as the gold standard, as these data were entered during rather than after the chest camp.

We assessed **completeness** by calculating ratios and 95% confidence intervals of all CAPI individual records per chest camp indicator amongst total PAPI individual records.

***Completeness*** =    *No. patient records created in CAPI tool per indicator*      *No. patient records created in PAPI tool* (1)

**Accuracy** was assessed by identifying the number of CAPI chest camps with the exact same (i.e., correct) number of data points as those appearing in the respective PAPI chest camp, also per chest camp indicator. For example, a chest camp event X was assessed as accurately entered in the CAPI tool for each indicator if, e.g., 100 individuals were indicated as presumptive in the PAPI tool and 100 individuals were marked as presumptive in the CAPI tool. If the CAPI tool showed 99 presumptive individuals for chest camp X and the PAPI tool showed 100 presumptive individuals for chest camp X, the CAPI records were considered inaccurate.

**Consistency** was assessed by calculating means and 95% confidence intervals of observed differences for each chest camp indicator (subtracting CAPI from PAPI) per van.

***Consistency*** = *mean(PAPI records per indicator – CAPI records per indicator) (per van)* (2)

To accommodate for data dependencies which could be expected from van teams screening in specific geographic clusters, all confidence intervals were calculated using STATA’s surveyset command identifying the van as the clustering factor.

### 2.7. Qualitative Analysis

To assess user acceptance of the CAPI tool, we used a thematic coding framework following the principles of user-friendliness, efficiency, and safety. The local consultant used the English translation of the Urdu transcriptions for analysis, and coded topic-specific responses into positive, negative, and neutral views.

### 2.8. Ethical Permission

Ethical approval was provided for quantitative and qualitative data collection and analysis by Pakistan National Tuberculosis Program’s Institutional Review Board on 20th January 2020 with approval code F number IRB-CMU-2020-10. Consent to participate in focus group discussions was sought and provided by all DFS participants prior to data collection. Patient-level data were anonymized before analysis in both CAPI and PAPI datasets by discarding personal identifiers from the study database.

## 3. Results

### 3.1. CAPI Completeness by TB Chest Camp Indicator

Of 142 chest camp events in the PAPI database and 147 in the CAPI database, a total of 140 events were found in both and therefore used for quantitative analysis. These 140 chest camp events were conducted in a total of 121 union councils located in 36 tehsils of Punjab and Khyber Pakhtunkhwa provinces (Figure 1). Exact locations were captured for 80 (57%) of the 140 chest camp events, and all of the remaining 60 events could be mapped manually using chest camp venue data entered into the CAPI tool.

In the PAPI dataset, 6876 individuals were registered as attendees across the 140 chest camp events, and the CAPI tool registered 6922 individual patient records (Figure 2). For only one other indicator, people with presumptive TB, the CAPI tool registered more patient records than PAPI: 3541 compared to 1505 individuals for CAPI and PAPI, respectively.

The CAPI/PAPI completeness ratios for all remaining indicators were less than 1.0, meaning that fewer records were entered digitally (via CAPI) than on paper (Table 2). People verbally screened and laboratory tests were both approximately 0.70, whereas completeness ratios for people with abnormal chest X-ray results and those diagnosed with pulmonary bacteriologically confirmed (B+) TB were 0.92 (95% CI: 0.21–1.64) and 0.96 (95% CI: 0.32–1.61), respectively (Table 2). Among all indicators, the number of people with any TB diagnosis (all forms) reported via CAPI had the lowest completeness ratio at 0.63 (95% CI: 0.29–0.97). As CAPI registered more individuals for chest camp attendees and people with presumptive TB, these indicators had ratios above 1.0 at 1.01 (95% CI: 0.90–1.12) and 2.35 (95% CI: 1.30–3.41), respectively.

### 3.2. CAPI Consistency

**Figure 3:**
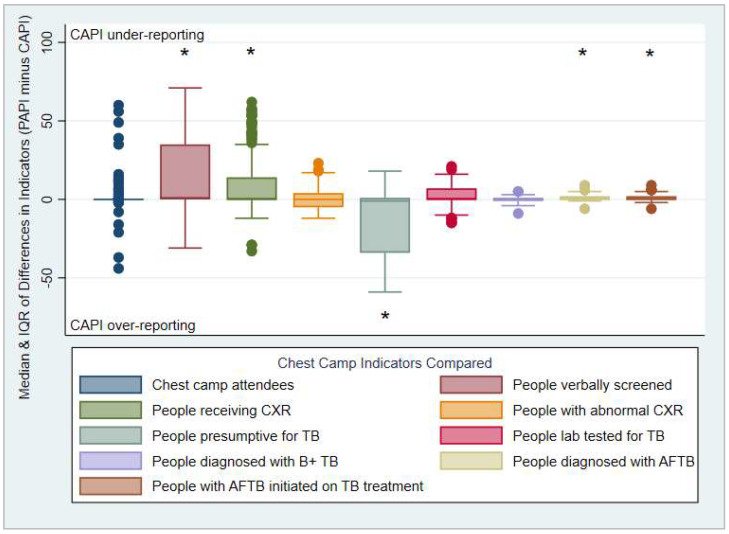
Median and interquartile ranges of mean differences between PAPI and CAPI chest camp indicators. * Significant difference (*p* < 0.05) in mean values reported between PAPI and CAPI.

Figure 3 presents the medians and interquartile ranges of the mean differences in CAPI and PAPI records. Table 2 and Figure 3 show that on average, verbal screening, chest X-rays, and all forms TB cases diagnosed and initiated on treatment were significantly under-reported via CAPI compared to PAPI, while presumptives were significantly over-reported in CAPI records. Significance tests of mean differences in indicators between PAPI and CAPI datasets are presented in Supplementary Table S1.

**Figure 4:**
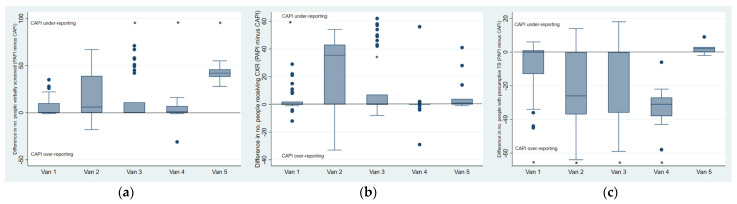
Consistency of CAPI and PAPI indicators Per van (PAPI-CAPI). (**a**) People verbally screened; (**b**) people receiving chest X-ray; (**c**) people identified with presumptive TB. * Significant difference (*p* < 0.05) in mean values reported between PAPI and CAPI.

We also explored van-level mean PAPI-CAPI differences in consistency of all chest camp indicators. For indicators in which the overall agreement between PAPI and CAPI varied largely across chest camp events (e.g., a large variance of mean differences was observed as shown in Supplementary Table S1) we calculated van-level mean differences and confidence intervals through which substantial differences in reporting could be observed (Figure 4a–c, Supplementary Figure S1a–c). Amongst those verbally screened, it is apparent that the overall under-reporting of CAPI occurred largely amongst vans three, four, and five, as vans one and two reported more consistently through CAPI. For people receiving chest X-rays, CAPI under-reporting also occurred the least in van one, although 95% confidence intervals for all vans overlap. CAPI over-reporting of presumptives occurred in all vans except for van five, which reported this indicator with a high level of consistency, significantly more so than all other vans.

### 3.3. CAPI Accuracy over Time

**Figure 5:**
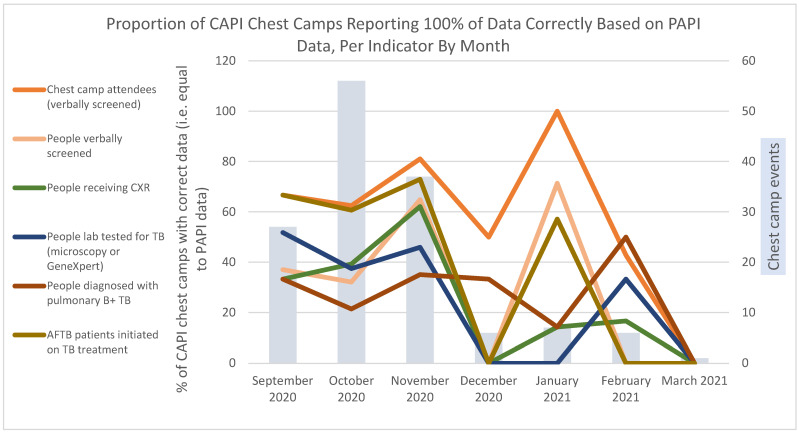
Proportion of CAPI chest camps reporting 100% of data correctly based on PAPI data, per indicator by month.

To assess accuracy of CAPI with respect to PAPI, we studied the proportion of chest camp events in which TB indicator values were exactly the same for CAPI and PAPI reports, by month. From September through November 2020, indicators maintained consistent levels of accuracy relative to each other. There were slight improvements between October and November 2020 (Figure 5). CAPI accuracy dropped to zero percent in December for all indicators except for chest camp attendees, pulmonary B+ diagnoses, and people with presumptive TB (not shown). These showed increases from 14.0% in October to 50.0% in December when chest camp activities and use of the CAPI tool slowed down. Compared to all other indicators, the number of chest camp attendees showed the highest levels of accuracy in all months, ranging between 42.3% and 100%. TB patients who had initiated TB treatment showed relatively high consistency in the first three months (59.6–73.0%); no consistent patterns were observable between January and March 2021. People receiving CXR consistently showed the lowest levels of accuracy in the CAPI dataset for all months except for January and February, when laboratory testing, verbal screenings, and all forms of TB (AFTB) treatment initiation showed lower accuracy.

### 3.4. User Acceptance

Four aspects of user acceptance of the CAPI tool were assessed in three focus group discussions held with the District Field Supervisors (DFS), the primary users of the tool. These aspects are the tool’s ease of use and usefulness, the tool’s effects on workload and efficiency, and safety concerns regarding using CAPI tools.

### 3.5. Ease of Use

The vast majority of the DFS found the CAPI tool to be generally easy to use after an initial acclimation period. While there were DFS who immediately found the tool to be user friendly, some others took a few months to become comfortable making entries at the speed required in chest camps. It was acknowledged in the focus groups that the digital literacy of the group varied considerably prior to use of the tool, and those who had little experience using digital devices seemed to struggle the most when training on and acclimating to the tool in the field. Even users with limited previous digital experience, however, were ultimately able to manage using the tool adeptly. All DFS in the focus group discussions claimed that it was quite user friendly.

### 3.6. Perceived Usefulness of CAPI

Many DFS identified useful components of the CAPI tool. On top of being able to improve efficiency of data entry, the DFS claim that in the event of loss or damage to paper based records, it is valuable to have the data backed up digitally. DFSs also found it practical to have access to all their digital data entry records on the web-based platform when they were asked to recall or share data from previous chest camps, which the CAPI tool allows them to do. Several of the DFS also shared that the tool helped to improve their digital literacy.

### 3.7. Effect on Workload and Efficiency

Data collection and patient registration in the Mercy Corps chest camps are currently completed through a hybrid process using PAPI and CAPI tools. As the CAPI tool has been added, instead of replacing PAPI, it created more work for the DFS during the data entry process. Many DFS vocalized that the increased workload from this hybrid model was at times burdensome and reduced their efficiency during camps, or that they were less able to give chest camp participants the same amount of attention before CAPI. Some DFSs appreciated how the CAPI tool’s automated functions can improve the workflow and organization of other components of chest camp. This includes the tool’s function of flagging presumptive patients based on symptom screening. DFS discussed that less time is now needed to analyze PAPI forms to identify patients for clinical follow up, as all presumptives are flagged in the CAPI tool. Although many DFS mentioned that the CAPI tool added time to their work process, they pointed out efficiency gains in producing monthly reports, which could eventually be dropped if CAPI technology is fully adopted.

### 3.8. Safety of Using CAPI Tools

The DFSs largely found that the use of digital tools in the field has not posed any physical safety issues to them, nor increased their risk of being targeted for robbery. They do, however, voice concerns about the comfort level of some community chest camp participants with the CAPI tool, who have on occasion seemed anxious about logging their personal data in a digital database following recent reports about local COVID-19 patients being traced and isolated.

One DFS in the focus group discussion did present some concern about data safety, claiming that in any sector, digital data can be hacked and misused. Most, however, did not share his concern and felt that having a digital repository backing up the paper documents has only served to create a safety net for the possibility of paper files getting lost or damaged.

## 4. Discussion

Digital data collection tools are increasingly used to monitor and evaluate the effectiveness and success of TB interventions. Although transitioning from paper to digital data collection can improve data quality and timeliness, there is limited documentation on how this can be operationalized in resource-limited settings. This paper has identified operational barriers which could hamper the uptake of digital tools and has quantified differences in completeness and accuracy between PAPI and CAPI data. Data quality of the CAPI tool was assessed from the perspectives of completeness, accuracy, and consistency with respect to PAPI records. CAPI completeness varied by month, increasing slightly over the first three months and tapering off through the next four months. DFSs reported an increase in the acceptance of CAPI after training and practice, consistent with other CAPI evaluations [13,14,18,22]. The tapering of CAPI data entry and diminution of accuracy over time was likely due to the suspended mandatory use of the tool pending a policy decision beginning in November 2020.

### 4.1. CAPI Data Quality

The relatively high degree of completeness of data reported through CAPI demonstrates that users were capable of utilizing the tool as intended (number of chest camp attendees (101%), people with abnormal chest X-ray (92%), people presumptive for TB (235%), and people diagnosed with B+ TB (95%)). The reason for apparent over-reporting of presumptives in CAPI was due the CAPI tool’s programmed logic: when any symptom was indicated, an individual was identified as presumptive as specified by Mercy Corps operational protocol. In practice, however, DFSs used a more specific definition to mark someone as presumptive based on the verbal screen, which was captured in PAPI chest camp records. In this case, CAPI data more completely represent the number of presumptives based on the definition set according to Mercy Corps policy, adding evidence to other studies reporting improved data completeness due to programmable logic in CAPI [3,14,17]. However, because a more specific definition was applied to identify a person with presumptive TB in practice, the data captured by the two tools are not perfectly comparable but rather reflect two different definitions of presumptive TB—that is, the presence of any TB symptom (CAPI definition) versus a more nuanced clinical definition applied by the interviewing medical officer who either discharged the participant from further evaluation or invited them to submit sputum and receive a chest X-ray (PAPI definition).

Indicators showing lower completeness such as chest X-rays (83%), lab tests (72%), and AFTB patients initiated on treatment (63%) may have reflected field staff data priorities. These fields were not mandatory in the CAPI tool, and were often skipped when the data input would have been equivalent to a zero (i.e., the patient did not receive a CXR or was not lab tested), or for perceived lower priority TB results (i.e., a clinical as opposed to a pulmonary B+ TB case). DFSs were pressed for time during chest camps, and most data entry for stages beyond verbal screening was performed after chest camp hours. In light of the fact that DFSs were held accountable for complete PAPI records but not CAPI, the observed volume of incomplete CAPI data seems a logical outcome of balancing time available, and the double burden of PAPI and CAPI data entry during busy periods. This prioritization of PAPI when accountability of CAPI data entry was no longer enforced also helps to explain the decreasing accuracy of chest camp level indicator totals over time.

### 4.2. Training and Supportive Supervision

Significant differences in consistency between van teams suggest that team practices are directly associated with data quality. Other studies have shown that CAPI users typically received at least one to two weeks of training on the CAPI tool, either in one stretch or through a series of trainings and periodic supportive supervision [19,28,31]. As our van teams received one to two days of training and limited supervision, this suggests that the training may have been insufficient for all to use the CAPI tool and protocols correctly. Another CAPI evaluation study documented that CAPI users struggled to switch between multiple data entry tools during data collection [19]. The DFSs also struggled to fill out CAPI and PAPI tools for all chest camp participants, leading them to prioritize PAPI data entry first. Therefore, while implementers making the shift from paper to digital data entry may view the double system as a safe temporary solution, it is not acceptable for many users.

### 4.3. Shifting from Hybrid (CAPI & PAPI) to CAPI

DFSs with a variety of digital literacy backgrounds described CAPI as practical, user-friendly, and enjoyable. These attitudes and their ability to use CAPI support other studies’ conclusions that users do not need to be familiar with digital technology to use CAPI effectively and that trainings should accommodate users with varying degrees of digital literacy [31,32]. However, in our setting, DFSs could not benefit from the normal efficiency gains offered by CAPI, as this was added onto routine PAPI data entry. DFSs indicated that data entry is their most time-consuming task, that they would welcome a full transition to CAPI, and that they would view the CAPI tool more favorably once the double data entry in PAPI and CAPI is eliminated.

Mercy Corps stated that once users show overall proficiency with CAPI, it can then replace the PAPI tool, which in similar studies has led to improved efficiency, replacing the arduous backend process of aggregating all paper data forms into a single excel file on a monthly basis and filling in both CAPI and PAPI forms during chest camps [19]. Unfortunately, it may be challenging for DFS users to demonstrate proficiency with CAPI while entry in both tools is still in effect, and while PAPI is still prioritized for accountability monitoring. For this reason, a CAPI tool for which users are primarily accountable and which can work fully offline is a necessary investment for implementers in low-Internet-access settings who wish to go fully digital, as described in TB case finding field manuals and evaluations of CAPI tools requiring matching between individual records [24,28,33]. However, working fully offline in this setting requires the use of individual barcodes, QR code, or patient ID to match data from the screening, sputum collection, chest X-ray, and laboratory/treatment “stages,” which requires financial investments and potential updates to the chest camp workflow. Furthermore, switching to a full CAPI-only data entry usually leads to temporary dips in data quality, but with timely supportive supervision, these gaps can be addressed and users can adapt [28].

### 4.4. Benefits of CAPI: Location Capture and Timeliness of Data for Decision-Making

There are considerable analytical benefits to collecting TB chest camp data using CAPI. Exact locations for all chest camp event data entered through CAPI were captured and available in near real-time. Having the data and locations digitally available as soon as the CAPI data were uploaded to the server allows implementers to understand where demand for TB services is located, and how TB burden is distributed geographically (i.e., where there seem to be “hotspots”) [34]. This enables implementers to respond to need for TB services more efficiently, an integral step in helping to interrupt TB transmission in a country with the 5th highest TB burden globally [35].

### 4.5. Limitations

There were several limitations in implementing the hybrid PAPI/CAPI data collection model effectively. The double workload of PAPI and CAPI data entry reduced the motivation to complete CAPI data entry, which was compounded by limited on the ground training M&E support, and a contractual issue caused CAPI data entry to taper out at the end of 2020. With more physical involvement, it may have been easier to address users’ concerns about the CAPI tool, and to improve the fit of the CAPI tool design to accommodate chest camp workflow, by introducing a scannable unique individual identifier. Furthermore, the hybrid approach necessitated identifying PAPI as the gold standard against which to compare CAPI data, but due to the limited M&E resources, we were unable to assure the quality of the PAPI data aggregated at the chest camp level.

## 5. Conclusions

Implementation of CAPI in low Internet access TB ACF settings is feasible, as demonstrated by high data completion during the first two months, high completeness by individual vans, and positive overall user feedback. The observed gaps in data quality and user dissatisfaction about the workload highlight two key takeaways. First, introducing digital technology is ideally accompanied by several key resources: record linking technology, clear protocols, supportive supervision, and data mentoring. Secondly, CAPI/PAPI hybrid models should be temporary until a full CAPI model is possible, even if rolled out for individual screening teams one by one. However, to make this transition, accountability for users must be placed on completion of digital, rather than paper tools.

Using devices with offline, location capturing, and record matching functionalities should greatly increase the scope of geographic areas in which CAPI can be used for TB ACF data collection, which is particularly relevant for low-Internet-access settings found throughout Pakistan and many other countries. Expanded use of CAPI in TB ACF settings and data sharing with NTPs will enable NTPs to consolidate ACF results into a single central geospatial repository, thereby collecting local estimates of TB prevalence measured at the community level. This will help TB programs to identify areas where under-detection or reporting is likely by comparing facility-based and ACF positivity rates and numbers needed to screen, and to help steer ACF activities in a more timely and evidence-based manner.

## Figures and Tables

**Table 1:** Individual-level data captured in both PAPI and CAPI tools aggregated to chest camp level for quantitative analysis.

Individual Level Data (i.e., Chest Camp Indicators)	Data Description
Personal identifiers	Name, date of birth, sex, village, contact information
Presence of symptoms	Cough, cough duration, hemoptysis, fever, night sweats, unintended weight loss, chest pain
TB history or TB contacts	Any TB history or close contacts with TB
Chest X-ray type	Analog or digital chest X-ray
Chest X-ray result	Normal or abnormal
Sputum specimen collected	Yes/No and if yes, specimen type
Type of laboratory test administered	Microscopy or polymerase chain reaction (PCR)
Laboratory test result	Presence of bacteriological confirmation and rifampicin resistance if PCR used
Final diagnosis made	Pulmonary bacteriologically positive (B+) TB/extra pulmonary TB/No TB

**Table 2:** Completeness of CAPI records per TB active case finding process indicator.

	CAPI Completeness Ratio (CAPI/PAPI)			
			**95% CI**	
**TB Active Case Finding Process Indicators**	**Ratio**	**Median**	**LB**	**UB**
Chest camp events				
Chest camp attendees	1.01	1.00	0.90	1.12
People verbally screened for TB	0.71 *	0.98	0.46	0.96
People receiving CXR	0.83 *	1.00	0.68	0.97
People with abnormal CXR	0.92	1.00	0.21	1.64
People presumptive for TB (based on 1 or more: CXR, symptoms, TB history)	2.35 *	1.11	1.30	3.41
People lab tested for TB (microscopy or GeneXpert)	0.72	0.91	0.26	1.18
People diagnosed with B+ TB	0.96	0.00	0.32	1.61
People diagnosed with AFTB	0.63 *	1.00	0.29	0.97
AFTB patients initiated on TB treatment	0.64 *	1.00	0.29	0.99

* *p* < 0.05.

## Supplementary Materials

The following supporting information can be downloaded at https://www.mdpi.com/article/10.3390/tropicalmed7080201/s1, Table S1: Means and mean differences in chest camp event indicators (PAPI—CAPI), Figure S1: Consistency of CAPI and PAPI indicators per van (PAPI—CAPI).
