# Supplementary material for: Factors Affecting the Transition from Paper to Digital Data Collection for Mobile Tuberculosis Active Case Finding in Low Internet Access Settings in Pakistan
Source: Trop Med Infect Dis. 2022 Aug 22;7(8):201. doi: 10.3390/tropicalmed7080201 (PMC9415978; doi:10.3390/tropicalmed7080201)
Supplement: Supplementary file 1 [file tropicalmed-07-00201-s001.zip › tropicalmed-1696941-supplementary.pdf]

Supplementary File

For all indicators except for chest camp attendees and people presumptive for TB, the average numbers reported in the CAPI dataset were lower as compared to the PAPI dataset (Table S1). The largest mean differences were found in people verbally screened (14.2) and presumptives (-14.5), also showing large variance (433.4 and 395.5 respectively), driven by the much larger variance of CAPI indicator means relative to PAPI. Although the average difference in the number of people attending chest camps was relatively small between PAPI and CAPI reporting (49.1 vs 4.4; diff = 0.33), the variance was very high (850.5) due to a large number of statistical outliers, which was also observed for number of people receiving chest x-rays. Only people verbally screened showed a statistically significant although very wide 95% confidence interval (1.0 – 27.5).

Table S1. Means and mean differences in chest camp event indicators (PAPI- CAPI).

	PAPI		CAPI		PAPI - CAPI Difference			
	Mean	Variance	Mean	Variance	Mean Difference	95% CI		Mean Difference Variance
Chest camp attendees	49.1	127.2	49.4	954.2	-0.3	-5.8	5.2	850.5
People verbally screened	49.1	127.2	34.9	444.1	14.2	1.0	27.5	433.4
People receiving CXR	44.2	143.3	36.5	1095.5	7.7	-0.4	15.5	1012.7
People with abnormal CXR	7.8	30.2	7.2	37.3	0.6	-5.2	6.4	59.3
People presumptive for TB (based on CXR, symptoms or history)	10.8	19.7	25.3	432.1	-14.5	-26.3	-2.9	395.5
People lab tested for TB (microscopy or GeneXpert)	8.8	21.2	6.3	28.2	2.5	-2.4	7.3	47.6
People diagnosed with pulmonary B+ TB	0.9	1	0.87	2.1	0.04	-1.6	0.6	3.0
People diagnosed with AFTB	2.5	3.3	1.6	3.6	0.9	-0.5	2.3	3.5
AFTB patients initiated on TB treatment	2.5	3.3	1.6	3.7	0.9	-0.5	2.3	3.5

Substantial variation in data consistency between CAPI and PAPI was observed between vans. Figure 1a–c show 95% confidence intervals around the mean differences in CAPI and PAPI records by van, for the indicators presented in the main document Figure 4a-c.

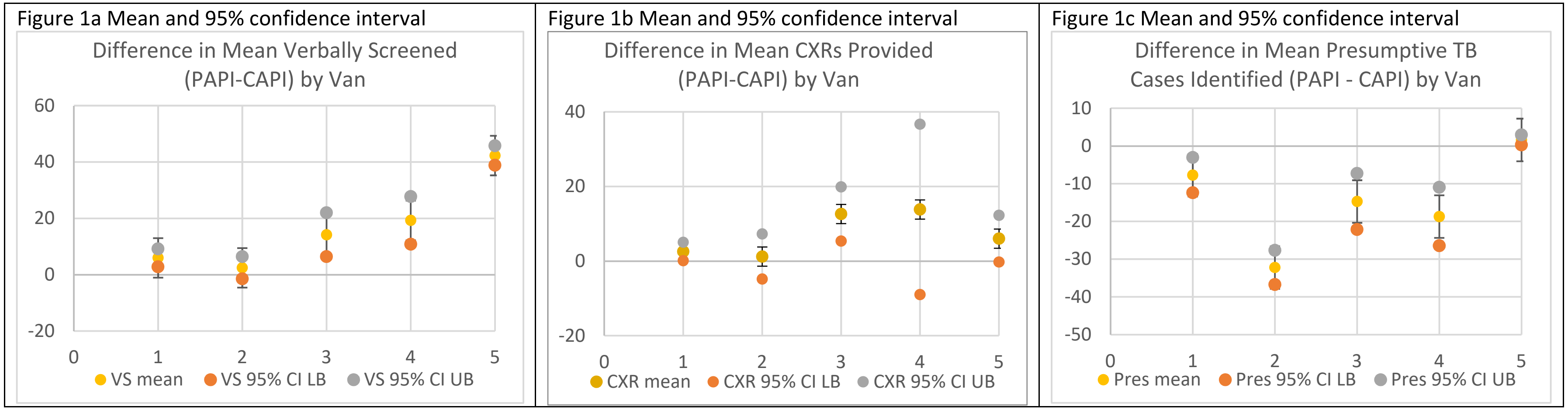

Figure S1. Consistency of CAPI and PAPI Indicators Per Van (PAPI- CAPI).
